# *Spiculopteragia asymmetrica* infection in *Cervus elaphus* from Iran

**Published:** 2014

**Authors:** Mohammad Reza Youssefi, Seyed Hossein Hoseini, Iraj Mobedi, Seyed Mohammad Hosseini, Behrang Ekrami

**Affiliations:** 1 *Department of Pathobiology, Faculty of Veterinary Medicine, Islamic Azad University, Babol Branch, Babol, Iran; *; 2 *Department of Parasitology, Faculty of Veterinary Medicine, University of Tehran, Tehran, Iran; *; 3 *Department of Parasitology and Mycology, Faculty of Public health, Tehran University of Medical Science, Tehran, Iran; *; 4 *Department of Animal sciences, Faculty of Agriculture, Islamic Azad University, Chaloos Branch, Chaloos, Iran.*

**Keywords:** Abomasum, *Cervus elaphus*, Iran, *Spiculoptenagia asymmetrica*

## Abstract

*Spiculopteragia asymmetrica* is a gastrointestinal nematode frequently found in the abomasum of cervids. During December and February 2010, two red deer were died in Semeskandeh sanctuary in Mazandaran province. Moreover, five live deer from mentioned area were treated by Ivermectin and collected feces of these animals were used for assessment helminthes infection by parasitological methods. Several nematodes were recovered in abomasums and in fecal samples of treated animals. Number of worms recovered from abomasums of two dead animals were 275 (90 male and 185 female) from the first one and 327 (102 male and 225 female) from the second. Based on morphological characteristics nematodes were diagnosed as *S.* *asymmetrica*. This is the first report of existence of *S.* *asymmetrica* from cervids in Iran.

## Introduction

*Spiculopteragia asymmetrica* is a gastrointestinal nematode frequently found in the abomasum of cervids including roe deer (*Capreolus capreolus*), red deer (*Cervus elaphus*) and fallow deer (*Dama dama*). It is rare in some domestic and sylvatic bovids and camelids primarily in the Palearctic and Eurasia.^1^^-^^3^ The history and geographic distribution of *Spiculopteragia* spp. in the world is not completely known. The first report of *S. spiculoptera* was from white tailed deer on Anticosti Island, Quebec, Canada, however, the origin of this parasite on the island has not been clarified. The historical geographic range of *Spiculopteragia* considered herein, apparently includes the Palearctic and Eurasia. However, the current geographic range has expanded through translocation of infected hosts. Consequently, *S. spiculoptera* has become established in New Zealand, Australia, and Argentina and *S. asymmetrica* in New Zealand, USA, Argentina and Turkey.^4^^-^^6^ The life cycle of* Spiculopteragia* spp.in sylvatic ruminants are direct, however, specific details of larval development and adult longevity are undetermined. Adults reside in abomasum, embryonated eggs are passed in feces, and the first through third larval stages are free-living. The prepatent period requires between two and three weeks.^7^

There is no report of *Spiculopteragia* species from cervids in Iran. This study described the existence and the morphology of *S. asymmetrica* in red deer.

## Materials and Methods

During December and February 2010, two *Cervus elaphus* were died in Semeskandeh sanctuary in Sari, Mazandaran province. These animals were autopsied. For the first deer infectious necrotic hepatitis and for the second one impaction was diagnosed as the cause of death. To determine the helminthes infection, the content of abomasum, small intestine and large intestine were washed away separately throw a sieve (mesh 100) and infestation rate was evaluated through counting the total worms. In the abomasums of both of deer several worms were recovered. Nematodes were cleaned with physiological saline and preserved in a solution of 70% ethanol. After cleaning, morphometric data were determined using a calibrated light microscope with ocular micrometer (Leitz, Wetzler, Germany) and a camera lucida (Carl Zeiss, Jena, Germany). Furthermore, five live deer from mentioned area were used for assessment of helminthes infection by parasitological examinations. For this purpose, each animal was treated by 0.2 mg kg^-1^ subcutaneous injection of Ivermectin 1% (Razak Pharmaceutical Co., Tehran, Iran) to purge intestinal worm. All collected fecal specimens were passed through a sieve and worms recovered as mentioned above. For determination of eggs in the feces, the fecal samples were collected and examined by flotation method with mixing saturated zinc chloride and saturated sodium chloride solution (Density = 1.52).

For confirmation of diagnosis, five male and five female nematodes were sent to US National Parasite Collection, Baltimore Avenue, Beltsville, USA. The specimen was deposited, as *S. asymmetrica* with the code number: USNPC, 104365. 

## Results

Based on morphological characteristics recovered nematodes were diagnosed as *S. asymmetrica* and all of the examined animals were infected only with this species. 

**Fig. 1:**
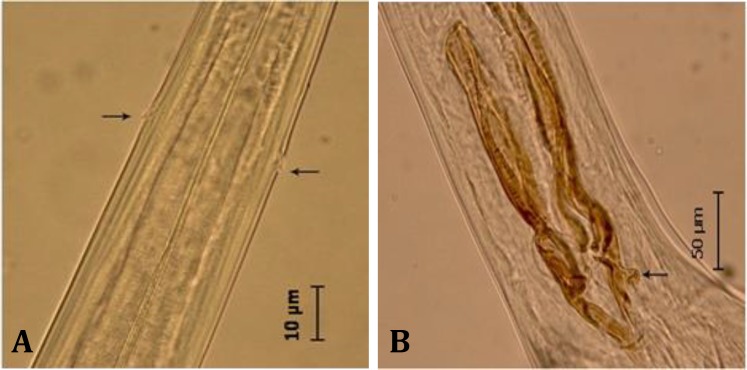
**A)** *Spiculopteragia* *asymmetrica*, anterior end, cervical papilla (Arrows); **B)** Spicules of *S. asymmetrica*, terminal nod (Arrow).

**Fig. 2:**
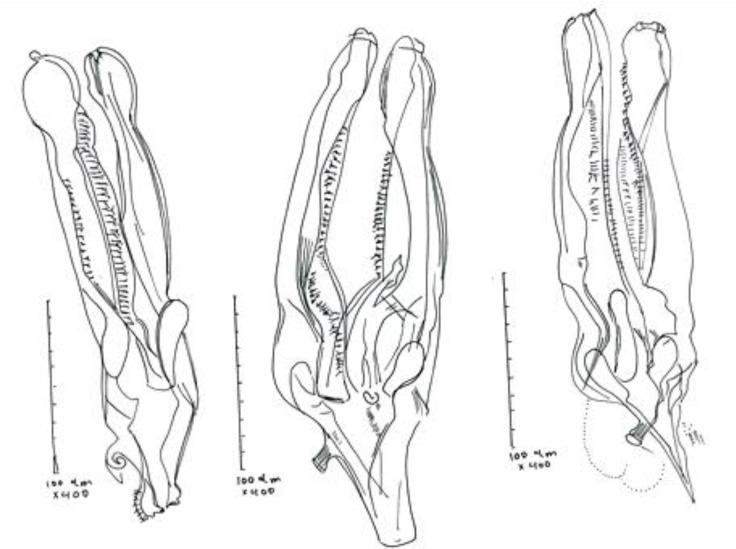
Spicules of *S.* *asymmetrica* in *Cervus elaphus* (Drawing by camera lucida).

Number of worms recovered from two dead animals were 275 (90 male and 185 female) and 327 (102 male and 225 female). In the fecal examination of treated animals a few number of worms (45.0 ± 5.0) were also recovered. Nematode descriptions are as follow:

 Male: They are small and thin. Length of body is 6.5 ± 1.5 mm. Anterior end without cuticular thickening. There are longitudinal ridges on cuticle. Cervical papillae are present (Fig. 1A). The two spicules are dark brown color and almost equal size (249.0 ± 15.0 µm). The spicules on their terminal part have a nod with size of 9.0 ± 1.0 µm, height of 15.0 ± 1.7 µm (Figs. 1B and 2). 

**Fig. 3:**
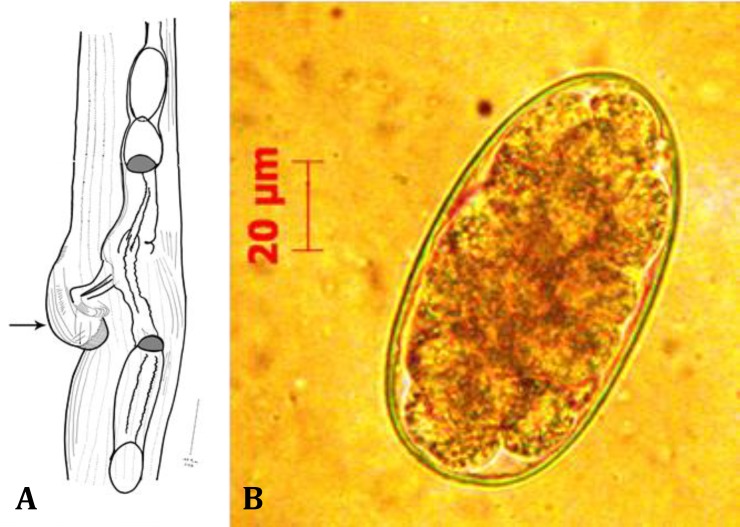
**A)** Female* S.* *asymmetrica;* vulva (Arrow), (Drawing by camera lucida); **B)** Strongyle type egg recovered from feces by flotation method

Female: Average size of female was 8.5 ± 2.0 mm. Vulva lies 2.5 to 2.7 mm from the end of tail (Fig. 3A). In the examination of fecal samples of treated animals, strongyle-type eggs were observed. The eggs are oval and have a length of 82.0 ± 7.0 µm and a width of 44.0 ± 5.0 µm (Fig. 3B).

## Discussion

*Spiculopteragia* is a parasitic nematode in many species of cervids and domestic animals in the world and it may be regarded as a pathogen in wild ruminants. Red deer and Roe deer are primarily main reservoir hosts of *Spiculopteragia* but domestic livestock can rarely get infected. The taxonomic status of *Spiculopteragia* is not clear and little is known about its pathology and epidemiology. The genus *Spiculopteragia *is similar to *Ostertagia *but differs in that males have no gubernaculum and their spicule have a nod at the posterior end.

There have been some studies for evaluation of parasitic infection in wild cervids and several species have been reported in this genus from America, Europe and Asia.^1^^,^^4^^-^^6^ Fruetel and Lankester reported *S. spiculoptera* from captive woodland caribou in Ontario, Canada.^7^^,^^8^ The first report of *S. asymmetnica* in the United States was from fallow deer on Little St. Simons Island, Georgia, USA.^9^ Subsequent records were from fallow deer from Kentucky,^10^ and Texas.^11^ Rossi *et al*. reported *S. spiculoptera *in the roe deer in Italy.^12^ Detected nematode species in roe deer in Turkey were identified as *S. spiculoptera *and *S. mathevossiani.*^6^ There is no study on helminths infection of red deer in Iran and also *Spiculopteragia *species are not previously reported. Based on morphological characteristics detected nematodes in red deer were diagnosed as *S. asymmetrica*. This is the first report of existence of *S. asymmetrica* from cervids in Iran. However, additional studies are needed to evaluate epidemiology and morphology of the parasite in wild life animals.
